# Electron Magnetic Resonance Study of Ni_50.2_Mn_28.3_Ga_21.5_ Powders

**DOI:** 10.3390/ma17174391

**Published:** 2024-09-05

**Authors:** Łukasz Dubiel, Bogumił Cieniek, Wojciech Maziarz, Ireneusz Stefaniuk

**Affiliations:** 1 Department of Physics and Medical Engineering, Faculty of Mathematics and Applied Physics, Rzeszow University of Technology, Powstancow Warszawy 12, 35-959 Rzeszow, Poland; 2 Institute of Materials Engineering, College of Natural Sciences, University of Rzeszow, Pigonia 1, 35-310 Rzeszow, Poland; bcieniek@ur.edu.pl (B.C.); istef@ur.edu.pl (I.S.); 3 Institute of Metallurgy and Material Science Polish Academy of Science, Reymonta 24, 30-059 Krakow, Poland; w.maziarz@imim.pl

**Keywords:** NiMnGa powder, Heusler alloys, electron magnetic resonance, spin-glass alloy, magnetic properties

## Abstract

In the present paper, we present an electron magnetic resonance (EMR) study of ${\mathrm{Ni}}_{50.2}{\mathrm{Mn}}_{28.3}{\mathrm{Ga}}_{21.5}$ powders obtained from melt-spun ribbons in the milling process. We registered EMR spectra in various temperatures at the X-band. In the EMR spectra recorded for the samples taken at the beginning of the milling process, the “training effect” was observed. After 2 h of milling, this phenomenon was no longer observed. To determine the basic EMR parameters, such as linewidth, resonance field, and asymmetry parameters, the experimental data were fitted using a single metallic Lorentz line. In high-temperature regions, we observed the influence of dispersion on the shape of the spectra, but as the temperature decreased, the asymmetry of line was reduced. The shift in the resonance field value at high temperatures and the temperature dependence of the linewidth below Curie temperature indicate that the investigated samples exhibited a characteristics of a spin-glass alloy.

## 1. Introduction

Ni-Mn–based alloys are an attractive group in the Heusler alloys family. In general, the magnetic moments in Ni-Mn-X (≈$4\mu_{B}$) largely originate from Mn atoms [1,2] and a small amount of magnetic moment equal to $0.3\mu_{B}$ related to *s*-like electrons from Ni. The contribution from the sp electrons of the X atoms to the magnetic moment is negligible [3]. In an off-stoichiometric composition, the extra atoms occupy sites in other sub-lattices, which leads to changes in distances between the same atoms [4,5,6,7]. Due to the hybridization between Ni and Mn atoms and the exchange interactions between Mn atoms mediated by Ni atoms, the change in symmetry results in changes in the alloy’s properties [8].

Ni-Mn-Ga alloys have been the subject of intensive studies due to the strong coupling exisiting between the structural ordering [9,10], the stoichiometry [11] of these materials, and their mechanical and magneto-mechanical properties, such as the giant magnetocaloric effect (MCE) [12,13,14] and large magnetoresistance (MR) [15]. Among these properties, the main interest lies in the magnetic field control of the shape memory effect, which was first observed by Ullako [16]. The ferromagnetic shape memory effect (FMS) [16,17] makes these materials very promising for applications in microelectronic devices [18,19,20]. Ni-Mn-Ga alloys in single-crystal form are the most well-studied and show promise in potential applications [21,22]. However, at the moment, using single-crystal materials in industrial large-scale applications is not possible because of cost and manufacturing limitations. On the other hand, relatively large magnetic FMS effects have been reported in melt-spun ribbons [23,24]. In polycrystalline Ni-Mn-Ga, the magnetic-field-induced strain might increase through the introduction of a crystallographic texture [25,26,27,28]. The increasing interest in additive manufacturing has a direct impact on the growing interest in polycrystalline Ni-Mn-Ga, including melt-spun ribbons and powders [28,29,30,31].

Electron magnetic resonance (EMR) is a very sophisticated tool that can be used to study magnetic properties and electron structure. Moreover, using EMR spectra, one can determine the magnetic ordering, phase transition temperatures, and spin dynamics of the investigated system [32,33]. Additionally, due to the high sensitivity of the EMR technique to local changes in the environment of magnetic ions, it is possible to obtain information about the magnetic properties of the material on a microscopic scale [34,35,36,37,38]. Nevertheless, in the literature regarding Heusler alloys, the amount of research based on the EMR technique is insignificant. In this paper, fir the first time, we use electron magnetic resonance (EMR) for ${\mathrm{Ni}}_{50.2}{\mathrm{Mn}}_{28.3}{\mathrm{Ga}}_{21.5}$ powders originating from melt-spun ribbons. Line shape analysis, fittings, and simulations of lines are performed and the origin of the EMR lines is discussed in detail. EMR spectroscopy is a very powerful technique, because the data can be used to identify the nature of the magnetic interaction and their evolution [39,40,41,42]. The results of the temperature dependence of base EMR parameters show a different behavior compared with the EMR spectra of NiMn-based ribbons [43]. What is new is that the results have enabled the use of Becker’s model of spin-glass alloys for powder samples.

## 2. Materials and Methods

The ${\mathrm{Ni}}_{50.2}{\mathrm{Mn}}_{28.3}{\mathrm{Ga}}_{21.5}$ alloy was prepared using induction melting from high-purity metals (>99.9%) in an argon atmosphere. The melt-spun ribbons were produced by ejecting molten alloys with argon overpressure (0.25 MPa) onto the surface of a copper wheel rotating at a linear speed of 25 ${\mathrm{ms}}^{-1}$. The powder was produced by mechanical milling of melt-spun ribbons in a vibration mill with a 50 mm diameter ball and under a 0.5 mm vibration amplitude for 8 h. The powder morphology, structure, composition, and entire preparation procedure were described in [44,45]. The samples used for research were taken during breaks in the milling process. Based on the milling time, the samples were labelled as NMG-05, NMG-1, NMG-2, and NMG-8 for 0.5, 1, 2, and 8 h of milling, respectively. The samples are listed in Table 1.

Electron magnetic resonance (EMR) measurements were performed using the Bruker ELEXYS E580 spectrometer equipped with the Bruker liquid N gas flow cryostat with the 41131 VT digital controller (Bruker Analytische Messtechnik, Rheinstetten, Germany) in the X-band (9.44 GHz). In the X-band, the spectra were registered using a standard super-high-Q resonator (ER 4123D) at a 100 kHz magnetic field modulation with an amplitude of 1 G.

The EMR parameters were obtained by fitting the theoretical curve to the experimental data and using OriginPro 2022b software (OriginLab, Northampton, MA, USA).

## 3. Results and Discussion

**Figure 1:**
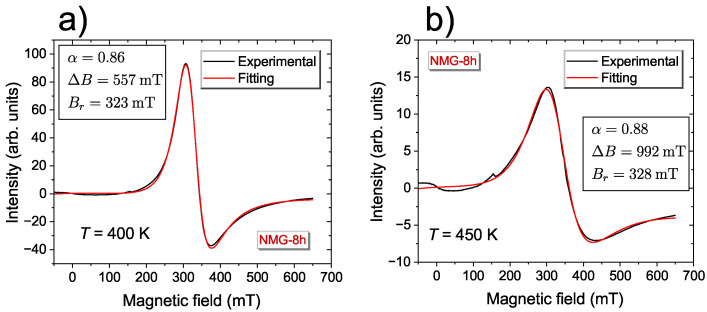
Representative EMR spectra of the derivative of the resonance absorption of ${\mathrm{Ni}}_{50.2}{\mathrm{Mn}}_{28.3}{\mathrm{Ga}}_{21.5}$ for the 8 h milling sample, measured at the X-band, showing their fitting using metallic Lorentz curves and fitting parameters at 400 K (**a**) and 450 K (**b**).

The EMR spectra as a function of temperature were taken in the temperature range of 100 K ≤T≤ 450 K. Figure 1 shows some of the selected spectra for NMG-8 registered at high-temperatures. These spectra contain a single, strong asymmetric line and the main reason for the asymmetry is the existence of a dispersive part in the EMR signal. The existence of the skin-depth effect [46] in metallic samples causes the appearance of dispersion in the EMR lines. The asymmetric lines recorded for samples with a high electrical conductivity can be fitted by the metallic Lorentz shape line, as described by Formula (1) [47]:

(1) $$\frac{dP}{dB}\propto \frac{d}{dB}\left(\frac{\Delta B+\alpha (B-B_{R})}{{\left(B-B_{R}\right)}^{2}+\Delta B^{2}}+\frac{\Delta B+\alpha (B+B_{R})}{{\left(B+B_{R}\right)}^{2}+\Delta B^{2}}\right),$$

where *B* is the induction of the magnetic field, α denotes the asymmetry parameter describing the proportion of absorption and dispersion parts in the EMR signal, ΔB is the EMR linewidth, and $B_{R}$ is the resonance field. For α=0 the EMR line is symmetric.

Proper milling of the powder and proper grain size resulted in the effective skin depth being better than the bulk sample, and, consequently, the contribution of the dispersion part in the EMR spectra was negligible. For NMG-8, the highest number of particles was in the range of (1–25) $\times \;10^{-6}$ m [44]. As a result, the spectra were more symmetrical compared with the bulk sample [38,48].That EMR signal could be assigned with ${\mathrm{Mn}}^{2+}$ ($3d^{5}$), with spin S=5/2. It was in agreement with earlier reports, which denoted that all magnetic moments in ${\mathrm{Ni}}_{2}\mathrm{MnZ}$ originated from Mn atoms and, generally, manganese atoms are very useful for in the magnetic properties of those materials [2]. A high concentration of 3d ions (Mn), caused broadening of the EMR signal and, as a consequence, the fine and hyperfine structure was unresolved [43,46].

During the decrease in temperature, the amplitude of the EMR line increased until the temperature was equal to T=350 K, which was the maximum value. As the intensity of the line increased, its asymmetry decreased, i.e., for temperatures of 450 K, the asymmetry parameter was α=0.89, while for T=300 K, the value of this parameter was 0, i.e., the observed line had a typical symmetric Lorenzian shape. The EMR line position in this region was independent of temperature.

**Figure 2:**
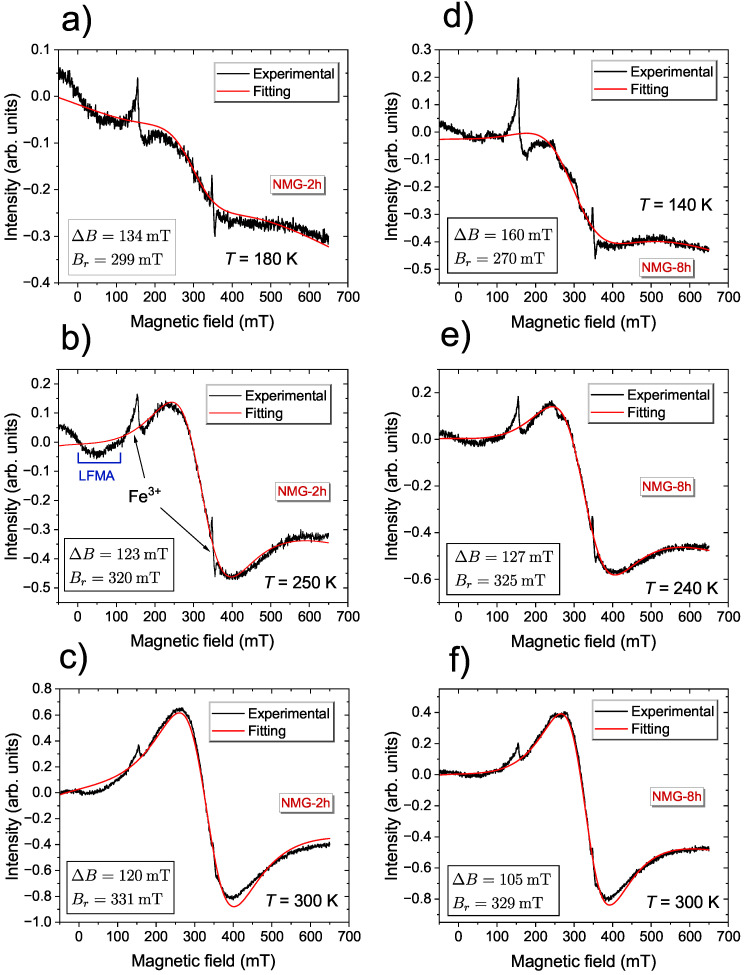
Representative EMR spectra of a derivative of the resonance absorption of ${\mathrm{Ni}}_{50.2}{\mathrm{Mn}}_{28.3}{\mathrm{Ga}}_{21.5}$ for different milling times: 2 h (**a**–**c**) and 8 h (**d**–**f**), measured at the X-band, showing their fitting using Lorentz curves and fitting parameters.

Figure 2 exhibits the EMR signals for selected temperatures for NMG-2 and NMG-8, registered at lower temperatures. A characteristic feature of these lines is their inclination across the entire field range. Typically, the intensity of a typical EMR line above the resonance field should asymptotically approach zero; however, behavior is not observed in this case. This shape of the line indicates that in this temperature range, there was a magnetic order in the samples. Another peculiarity for the EMR line at low temperatures, for both the NMG-2 and NMG-8 samples, was the two narrow peaks around 150 mT and 350 mT, which corresponded to the effective *g*-values $g_{1}\approx 4.3$ and $g_{2}\approx 2$, respectively. Because the intensity of the main EMR line decreased as the temperature decreased, the additional narrow lines became more visible. These additional lines could be assigned to the ${\mathrm{Fe}}^{3+}$ ion and the presence of these peaks could be explained by trace amounts of iron emerging from the milling process and the high sensitivity of the EMR technique. Many authors have demonstrated the ease of identifying ${\mathrm{Fe}}^{3+}$ ion impurities using electron magnetic resonance techniques [49,50,51]. In this temperature range, the main line fit well to a single, broad, symmetric Lorentz line.

**Figure 3:**
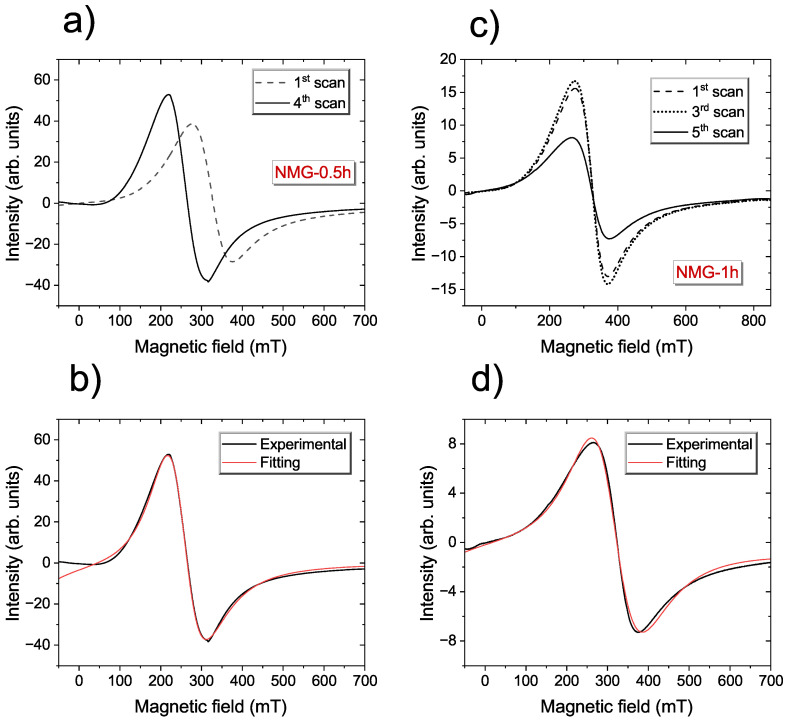
Cycle dependence EMR spectra scanned at room temperature for NMG-0.5 (**a**) and NMG-1 (**c**). The lower panel exhibits the fitting by using the metallic Lorentz shape line for NMG-05 (**b**) and NMG-1 (**d**), respectively.

Figure 3 presents the time evolution of the EMR signal for NMG-0.5 and NMG-1. The cycle dependency of the EMR spectra was evidenced by scanning the sample using a magnetic field between 0 and 700 mT several times and then recording the EMR lines. For samples taken in the initial phase of the grinding process, in the EMR spectra, a “training effect” was observed, i.e., the shape and line position changed during later repetitions of the EMR measurements at constant experimental conditions [52]. A similar behaviour of the EMR spectra was observed for Ni-Co-Mn-In flake ribbons below the Curie temperature [38], and the changes in EMR signal were connected to the coexistence of different magnetic phases at this temperature. In the remaining samples, where the grinding time was correspondingly longer, no relationship was observed between the shape of the spectrum and cyclic magnetic field scanning.

In the diluted magnetic alloys, the EMR linewidth increased linearly with the temperature, and this thermal broadening can be written by $\Delta B=a_{0}+bT$, where $a_{0}$ denotes the residual linewidth and *b* is a Korringa rate. Departures were noteed from the Korringa behaviour, i.e., departures from linear dependence where the observer in the more concentrated magnetic system underwent magnetic ordering [46,53]. In the spin-glass system, the linewidth dependence was more complex, and the simple Korringa relaxation was observed only in a paramagnetic state in a high-temperature region [53].

For spin-glass alloys, thermal broadening was proportional to 1/M(T), and if M(T) follows, according to the Curie–Weiss law, the thermally broadening linewidth is given by [54]

(2) $$\Delta B=\left(a_{0}-b\theta \right)+bT,$$

where $a_{0}$ is the residual linewidth, θ is the Curie–Weiss temperature, and *b* is the thermal broadening constant.

**Figure 4:**
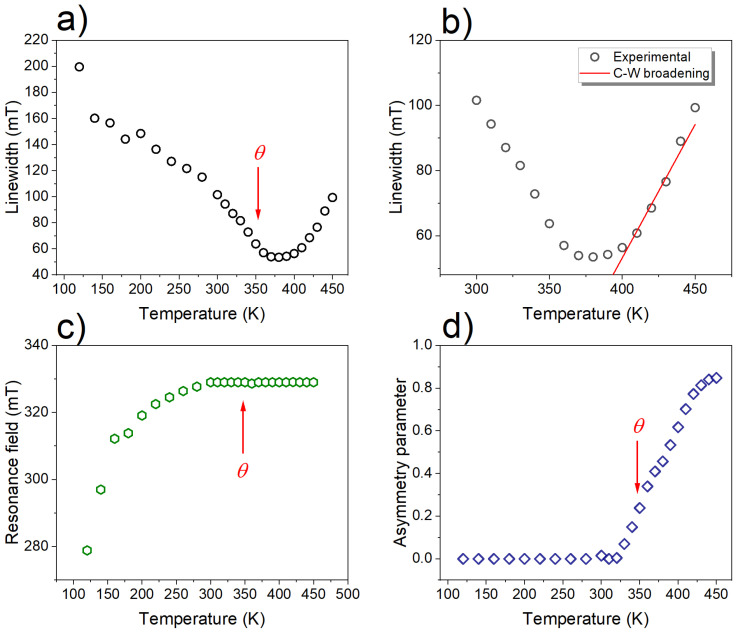
Temperature evolution of the basic EMR parameters for NMG-8: linwidth (**a**,**b**), resonance field (**c**), and asymmetry parameter (**d**).

The fit of Equation (2) for a high-temperature region, above θ, is shown in Figure 4b, where the residual linewidths are $a_{0}=139.5$ mT and b=8.2 mT/K.

The temperature dependence of the resonance field $B_{R}\left(T\right)$ is shown in Figure 4b. Within the temperature range of 300 K ≤T≤ 450 K, the resonance field is independent of temperature. This behaviour of the resonance field above $T_{C}$ is typical for spin-glass alloys, as reported by other authors [54,55]. Based on the formula $h\nu =g\mu_{B}B_{R}$, one can calculate the value of the effective *g* factor (*h* is the Planck constant, ν is the microwave filed frequency and $\mu_{B}$ is the Bohr magneton). Above 300 K, a positive *g* shift was observed (the shift with respect to the *g* factor for ${\mathrm{Mn}}^{2+}$ in an insulator [56,57]), and at this temperature range, the value of the *g* factor was close to 2.07. With future decreasing temperatures, the resonance field was still dependent on temperature and started to shift toward a low field.

**Figure 5:**
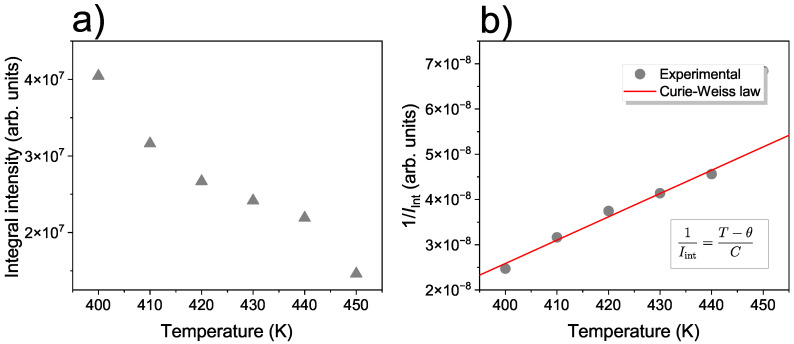
Temperature dependence of integral intensity of the EMR signal for NMG-8 (**a**) and inverse integred intesity versus temperature (**b**).

Based on the linewidth, it is possible to determine the integral intensity $I_{\mathrm{int}}$ of the EMR signal, which is proportional to the dynamic susceptibility $I_{\mathrm{int}}\propto \chi$ and is expressed by the formula [58]:

(3) $$I_{\mathrm{int}}=I\cdot \Delta B^{2}{\left(1+\alpha \right)}^{0.5}$$

where *I* denotes the peak-to-peak intensity of the EMR signal and α denotes the asymmetry parameters for the Lorentz line α=0 and for Dyson line α>0. Figure 5 presents the temperature dependences of the integral intensity and inverse integral intensity for NMG-8. As the inverse of $I_{\mathrm{int}}$ is proportional to the inverse susceptibility, according to the Curie–Weiss law, θ can be determined as the intercept on the temperature axis from a liner fit of $1/I_{\mathrm{int}}$ versus temperature. Using this method, θ is determined to equal 352 K.

**Figure 6:**
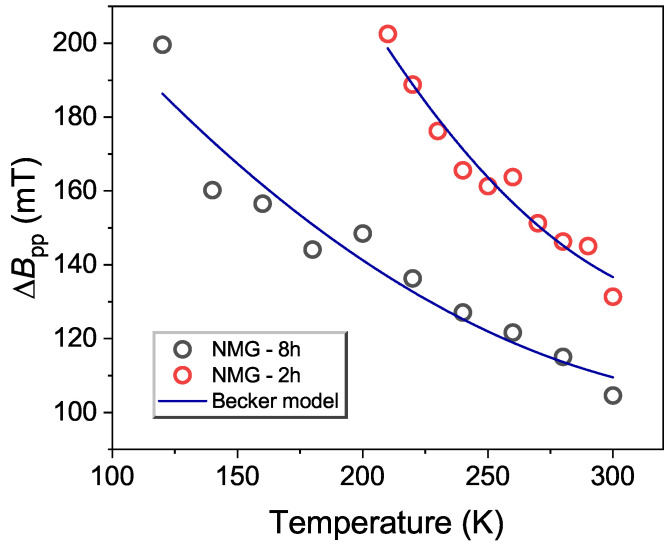
The temperature evolution of $\Delta B_{\mathrm{pp}}\left(T\right)$ for NMG-2 and NMG-8 using the Becker model.

To fit the peak-to-peak linewidth, below the Curie temperature, we used the function [54]:

(4) $$\Delta B=a_{0}+b^{\prime }{\left|\frac{T-T_{\min}}{T_{\min}}\right|}^{n}$$

where $a_{0}$ is the residual linewidth, $b^{\prime }$ is the thermal broadening constant, *n* is the exponent associated with the distribution of magnetization defined by Huber’s theory of linewidth near the critical temperature, and $T_{\min}$ corresponds to the temperature at which the linewidth reaches its minimum. The best fit of Equation (4) is shown in Figure 6 and the fitting parameters are collected in Table 2. It has long been reported that in spin-glass alloys, the value of the residual component of the linewidth is related to the strength of the crystal fields effect and the local magnetic moment perturbations through a demagnetization mechanism [54].The value of $a_{0}$ for both samples (see Table 2) suggests that the contributions of the demagnetization and crystal field effect in NMG-2 and NMG-8 are high.

## 4. Conclusions

In summary, we presented the EMR study of ${\mathrm{Ni}}_{50.2}{\mathrm{Mn}}_{28.3}{\mathrm{Ga}}_{21.5}$ powder. Two types of measurements were performed: cyclic measurements at room temperature and temperature measurements in a temperature range of 100 K ≤T≤ 450 K. In the cyclic spectra recorded for the samples taken at the beginning of the milling process, we observed a “training effect”.For each spectrum, the EMR signal was satisfactorily fitted using a single metallic Lorentz line. In high-temperature regions, we observed that dispersion influenced the shape of the spectra; however, as the temperature decreased, the asymmetry of the line was reduced. Based on fitting parameters such as intensity and linewidth, the temperature dependence of the integral intensity was determined. The Curie temperature, determined in this manner, was equal to 352 K. The shift in resonance field value at high temperatures and the temperature dependence of the linewidth below the Curie temperature indicates that the investigated samples had a spin-glass alloy character.

## Figures and Tables

**Table 1:** Investigated samples.

Label	Composition	Milling Time in Hour
NMG-05	${\mathrm{Ni}}_{50.2}{\mathrm{Mn}}_{28.3}{\mathrm{Ga}}_{21.5}$	0.5
NMG-1	${\mathrm{Ni}}_{50.2}{\mathrm{Mn}}_{28.3}{\mathrm{Ga}}_{21.5}$	1
NMG-2	${\mathrm{Ni}}_{50.2}{\mathrm{Mn}}_{28.3}{\mathrm{Ga}}_{21.5}$	2
NMG-8	${\mathrm{Ni}}_{50.2}{\mathrm{Mn}}_{28.3}{\mathrm{Ga}}_{21.5}$	8

**Table 2:** Investigated samples.

Label	$a_{0}$ (mT)	$b^{\prime }$ (mT)	$T_{\min}$ (K)	*n*
NMG-2	128	449	348	2
NMG-8	104	183	364	2

## Data Availability

The original contributions presented in the study are included in the article, further inquiries can be directed to the corresponding author.
